# Advanced motions for hexapods

**DOI:** 10.1177/1729881419841537

**Published:** 2019-04-09

**Authors:** Wei Cheah, Hassan Hakim Khalili, Farshad Arvin, Peter Green, Simon Watson, Barry Lennox

**Affiliations:** School of Electrical and Electronic Engineering, University of Manchester, Manchester, UK

**Keywords:** Legged locomotion, mobile robot, advanced motions, robot design

## Abstract

Advanced motions, which utilize footholds on walls, offer considerably more opportunities for hexapods in accessing confined environment. However, there has been no research on the practical application of such motions on a hexapod. These motions are kinematically viable for the standard hexapod design with three degrees of freedom per leg but the joint requirements have yet to be identified. This article presents the motion analysis for two forms of advanced motion, wall walking and chimney walking, to study the joint requirement for executing such motions. The analysis has been verified through a series of experiments demonstrating that a hexapod with a standard design is capable of executing advanced motions.

## Introduction

The advantages of legged robot locomotion over other types of propulsion have been widely discussed in the literature^1–3^ and have inspired competitions that try to replicate real-world scenarios where legged robots could be deployed for inspection and intervention missions. The DARPA Robotics Challenge (natural/man-made disasters),^4^ ARGOS (oil and gas refineries)^5^ and Eurathlon (urban and indoor environments)^6^ all provide demonstration areas to test the state of the art in legged locomotion.

**Figure 1.**
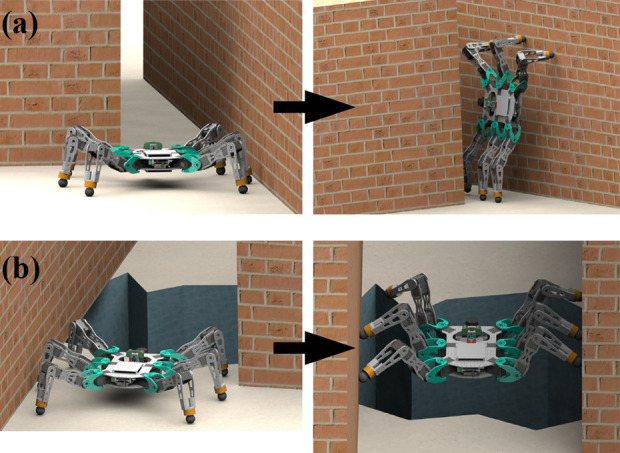
Advanced motions exploiting the robot’s full mobility for navigation through: (a) narrow space by wall walking and (b) a large hole by chimney walking.

All of the challenges mentioned above are largely concerned with two-dimensional (2D) motion, and navigating around obstacles that are, at most, of the same scale as the robot. Specifically, the areas being traversed accommodate the footprint of the legged robot. For example, doorways or stairwells are wider than the robot trying to navigate passed them. What has not been considered in any depth is the challenge of non-planar motion in cluttered spaces. Figure 1(a) shows the example of a hexapod manoeuvring through a gap that is narrower than its footprint by using the vertical surfaces of the gap to provide support. Figure 1(b) shows how three-dimensional (3D) motion can be achieved in certain situations.

This article is concerned with non-planar hexapod manoeuvres, which will be termed advanced motions and the scenarios shown in Figure 1 will be considered in detail.^7^ The movement shown in Figure 1(a) is termed wall walking and that in Figure 1(b), chimney walking. Such manoeuvres have not previously been considered in the literature.

The motions shown in Figure 1 are likely to impose additional requirements on the joints of a hexapod relative to two-dimensional manoeuvres. The aim of this article is to identify the effect of key design parameters on joint requirements for executing advanced motions. In particular, the relationship between the robot’s link lengths and motion plan and the joint requirements will be considered. It should be clearly understood that the work is based on a hexapod of standard design, and a key element is to investigate the extent to which an ‘off-the shelf’ hexapod is capable of executing advanced motions.

This rest of this article is organized as follows: First, the current state of art on typical hexapod motions and joint characterization approaches for hexapods are presented. Next, the joint requirements analysis for advanced motions is detailed followed by the mechanical design of the particular hexapod used in this work (Corin). The validation experiments and their results are then detailed. The final section concludes the work and an outlook to future work is detailed.

## Related work

**Figure 2.**
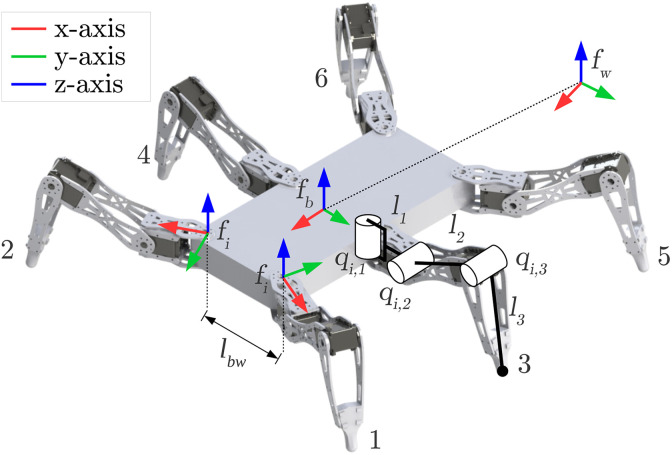
Summary of nomenclature for hexapods. Leg labels: odd number legs are on the left side ( i=1,3,5 ), while even number legs are on the right side ( i=2,4,6 ). Link labels: coxa link ( $l_{1}$ ), femur link ( $l_{2}$ ), tibia link ( $l_{3}$ ). Joint labels: coxa joint ( $q_{i,1}$ ), femur joint ( $q_{i,2}$ ), tibia joint ( $q_{i,3}$ ). Reference frames: world frame ( $f_{w}$ ), base frame ( $f_{b}$ ), leg frame ( $f_{i}$ ).

This section consists of two parts: the first provides a brief overview of typical hexapod movements, while the second provides an overview of link and joint selection approach for hexapods. The nomenclature used in this article is specified in Figure 2.

### Typical hexapod movements

The motion of a hexapod consists of two phases: the transfer phase, where a leg is transferred from one foothold to another, and the support phase, where the legs supporting the robot’s mass move the body in the desired direction.^1^ In virtually all cases, footholds are confined to the support (ground) surface. Footholds on irregular terrains such as grass^8^ or rocks^9^ can still be thought of as lying on the ground surface.^10^ This generalization can also be applied to inclined surfaces since, from a kinematic perspective, slopes have no effect on hexapod movements.

A small number of additional movements have been considered that utilizes non-ground (wall) surfaces. These are the climbing strategy used for surmounting large obstacles^11^ and moving through a narrow pathway with the aid of a semi-elliptical shell attached to the robot’s back.^12^ However, the use of wall surfaces is limited relative to what is proposed here.

### Link selection and joint characterization for hexapods

The key design parameters of a hexapod are its base width and length and the lengths of its leg links. These affect the joint requirements in terms of the range of motion, velocity and torque, and these in turn affect the design parameters. This results in a large number of interdependent parameters that must be selected during design. To reduce the parameter search space, a common approach is to first select the design parameters followed by the joint requirements or vice versa.

The selection of design parameters uses bio-inspired, pragmatic or optimization-based approaches, and sometimes a combination of approaches. Bio-inspired approaches base design choices on living creatures.^13–15^ Pragmatic approaches may, for example, base design decisions on the characteristics of the target deployment environment^14^ or on the torque limitations of the intended actuators.^16^ Optimization-based strategies may consider energy consumption,^17^ mobility^18^ or dexterity^19^ as the optimization metric.

Joint characterization of hexapods typically uses either static analysis^13^ or motion simulation,^8^ often with predefined link parameters. However, in the literature, both parameter selection and joint characterization are only ever considered in the context of two-dimensional manoeuvres. Clearly, an important consideration when considering non-planar hexapod motion is to consider how this affects link selection and joint characterization. In the next section, static analysis of advanced motions will be studied, in order to gain an understanding of the effect of design parameters on joint characterization. This will then allow the selection of appropriate design parameters and actuators for a hexapod capable of advanced motions.

## Design analysis

The effect of key design parameters on joint requirement for a hexapod with a standard design to execute advanced motions needs to be studied. This would enable the selection of actuators given the key design parameters, or vice versa, that meets the joint requirement for such motions. The key design parameter of interest here is the body width and leg’s link length, while the joint requirement is the maximum torque and range of motion required. This section characterizes both the motions of interest in this study (Figure 1) and studies the effect of link length and body width on joint requirement.

### Static analysis setup

**Figure 3.**
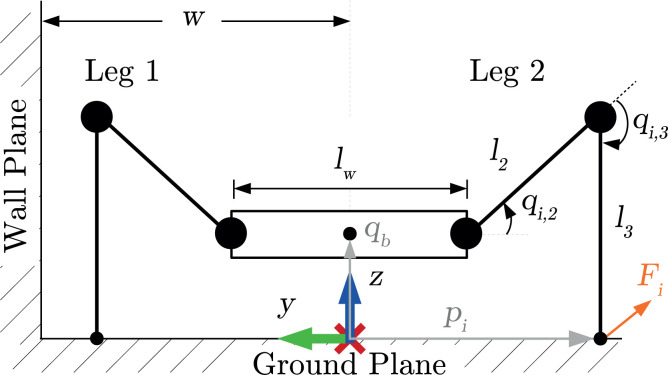
Summary of nomenclature for this analysis. Link labels: lumped body width ( $l_{w}$ ), femur link ( $l_{2}$ ), tibia link ( $l_{3}$ ). Joint labels: femur joint ( $q_{i,2}$ ), tibia joint ( $q_{i,3}$ ). Body pose ( $q_{b}$ ), foot contact ( $p_{i}$ ) and foot force ( $F_{i}$ ) are expressed with respect to the world frame.

The analysis of motions is based on the following assumptions:

1. The motions are discretized and semi-static.
2. The motion occurs only in the *y*-*z* plane (see Figure 3).
3. The total mass of the robot is assumed to act at the geometrical centre.
4. The tangential force lies on or within the friction cone edge.
5. The foot contact forces for multiple support legs on a single side are distributed equally for each leg.

From the second assumption, the robot model is simplified to have only two legs with two degrees of freedom (DoF), as shown in Figure 3. Gaits that have two support legs on either side of the robot are lumped together as a single leg for the force distribution analysis (discussed shortly). The coxa link, $l_{1}$ , is considered as part of the lumped body width, $l_{w}$ , hence


1 $$l_{w}=l_{bw}+2l_{1}$$

**Figure 4.**
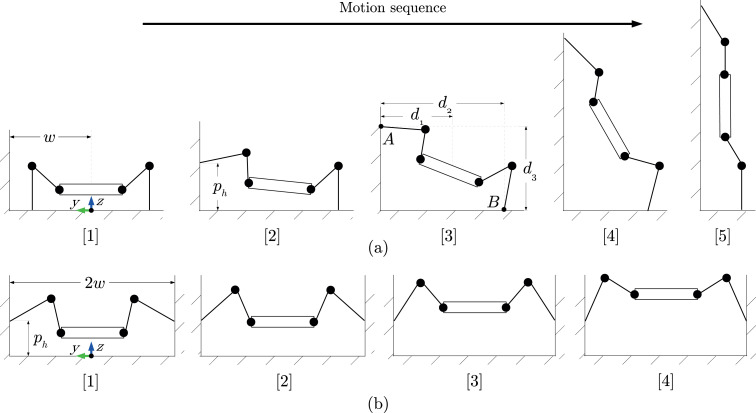
Illustration of the discretized motion sequence (from left to right), starting from ground to the final stance for (a) wall transition and (b) chimney walking. Each discretized motion is called a pose, numbered accordingly as $n_{s}=\left\{1,2,3,...,n_{d}\right\}$ .

The two characteristic motions considered here are called wall transition (Figure 4(b)) and chimney walking (Figure 4(a)). The wall transition, which refers to the motion of the robot rearing up sideways towards a wall, is analysed as a precursor to the analysis of wall walking (Figure 1(b)), as the demand on joint torque will be higher due to the longer horizontal moment arm between the robot’s geometrical centres to its feet.

The body pose, $q_{b}\in {ℜ}^{3}=[y,z,\theta ]$ , and foot contact, $p_{i}\in {ℜ}^{2}=[y,z]$ , are interpolated linearly between the start and end poses, except for the step where contact changes from the ground plane to the wall plane. With this, the joint angles for the legs can be determined using an analytical solution. The joint torque requirement for each leg is obtained as


2 $$\tau_{i}=J_{i}^{T}F_{i}$$

where $\tau_{i}\in {ℜ}^{2}$ , $J_{i}\in {ℜ}^{2\times 2}$ and $F_{i}\in {ℜ}^{2}$ are the *i*th leg’s joint torque, Jacobian and contact force, respectively. The total energy cost, E∈ℜ , is the power integrated over the motion sequences,^17^ which for the discretized case is


3 $$E=\sum_{n_{s}=0}^{n_{d}}\left(\tau_{n_{s}}^{T}{\dot{q}}_{n_{s}}\right)$$

where $\tau_{ns}\in {ℜ}^{2\times i}$ , ${\dot{q}}_{ns}\in {ℜ}^{2\times i}=q_{ns}-q_{ns-1}$ and $q_{ns}\in {ℜ}^{2\times i}$ are the joint vectors for legs 1 and 2 on torque, position and velocity (assuming a unit time to normalize the effect of joint velocity), and $n_{d}$ is the number of discretized poses.

### Force distribution

The contact force, $F_{i}$ , for both motions is calculated as follows.

#### Wall transition

The worst case scenario is when the robot uses the tripod gait where only one support leg is on the ground plane, while two support legs are in contact with the wall plane. Based on the sum of forces of the robot and moments at point B in Figure 4(a)-3


4 $$mg=2F_{1,z}+F_{2,z}$$

5 $$0=-2F_{1,y}+F_{2,y}$$

6 $$0=mg(d_{2}-d_{1})-2F_{1,z}d_{2}-2F_{1,y}d_{3}$$

where *m* is the robot mass, *g* is acceleration due to gravity and $d_{1}$ to $d_{3}$ are the effective distances (see Figure 4).

For no slip to occur, the tangential force for the legs in wall contact was selected to lie on the friction cone edge


7 $$F_{1,z}=\mu F_{1,y}$$

Substituting equation (7) into equation (6) yields


8 $$F_{1,y}=\frac{mg(d_{2}-d_{1})}{2(\mu d_{2}+d_{3})}$$

and the rest of the contact forces are


9 $$F_{2,y}=2F_{1,y}$$

10 $$F_{2,z}=mg-2F_{1,z}$$

#### Chimney walking

The torque demand for chimney walking is large, which is evident in the HyQ platform.^20^ Hence, it is assumed that the robot has a minimum of four support legs to reduce the joint torque demand. With four legs in support (two on each side), the tangential force distributed evenly across each leg is


11 $$F_{i,z}=\frac{mg}{4}$$

and the minimum normal force required to prevent slippage on each leg is


12 $$F_{i,y}=\frac{F_{i,z}}{\mu }$$

### Joint angle requirement

The maximum joint angle requirement is when the leg needs to move from ground to wall contact at its most constrained position, that is, the leg is tucked in as close as possible (Figure 10). The initial position of the foot is constrained structurally and will be identified following the selection of the link length for $l_{2}$ and $l_{3}$ . The foot is required to translate vertically until it reaches the wall contact distance height, $p_{h}$ , and then translate horizontally to make contact with the wall. The corresponding joint angles computed will be the range of motion required for the respective joints.

### Force distribution parameters

The parameters for the force distribution analysis are shown in Table 1. The ‘*’ implies that these parameters are dependent on link length parameters. The equations for these are available in Appendix 1.

**Table 1.** Parameters for the force distribution analysis.

Parameter	Symbol	Value	
		Wall transition	Chimney walking
Final body pose	$q_{b,\text{final}}$ (m, m, °)	[*,*,90]	[0,*,0]
Initial joint angles	$q_{i(\text{init})}$ (°)	$q_{1}=q_{2}=\left[20,-110\right]$	
		$q_{1}=[0,-10]$	$q_{1}=[*,*]$
Final joint angles	$q_{i(\text{final})}$ (°)	$q_{2}=[10,-10]$	$q_{2}=[*,*]$
Surface friction	*μ*	0.7	
Number of discretized pose	$n_{d}$	10	
Robot mass	$m_{\text{base}}$ (kg)	5.0	
Overall width range	$l_{ow}$ (m)	0.85 to 1.0	
Lumped body width range	$l_{w}$ (m)	0.3 to 0.4	
Link ratio range	$l_{2}:l_{3}$	(0.75 to 1.25):(1.25 to 0.75)	
Step size	*β*	0.05	
Translation distance	$d_{z}$ (m)	NA	0.15
Wall contact foot height	$p_{h}$ (m)	$2q_{b,z}$	$q_{b,z(\text{init})}+d_{z}/2$
Wall distance	*w* (m)	$w_{w}=0.03$	$w_{c}=0.36$

The lumped body width and body mass were assumed according to small-scale hexapods.^7^ A conservative *μ* was selected given the high friction observed from using rubber feet.^20^

The three parameters of interest in this study are the overall width, $l_{ow}$ , lumped body width, $l_{w}$ and link ratio, $l_{2}:l_{3}$ . The overall width, $l_{ow}$ , is the lateral width of the robot with its legs fully extended


13 $$l_{ow}=l_{w}+2(l_{2}+l_{3})$$

The range selected has been based on the required leg work envelope of at least 0.2 m. The link ratio, $l_{2}:l_{3}$ , has been selected which includes the ratio used on existing hexapods. For a given $l_{ow}$ , $l_{w}$ and $l_{2}:l_{3}$ , the length for $l_{2}$ and $l_{3}$ is obtained using equation (13).

The step size, *β*, is the resolution in which the parameters $l_{w}$ , $l_{ow}$ and $l_{2}:l_{3}$ are incremented. The translation distance, $d_{z}$ , is the distance the robot’s base travels for chimney walking. The wall distance for wall transition, $w_{w}$ , is the distance between the foot and the wall at the start (Figure 4(a)-1). The wall distance for chimney walking, $w_{c}$ , is the distance between the robot’s geometrical centre and the wall at the start (Figure 4(b)-1).

The wall contact foot height, $p_{h}$ , is the height from ground where the first contact is made with the wall (Figure 4(a)-2) and is dependent on the robot’s initial base height, $q_{b,z}$ . The effects of the parameters $w_{w}$ , $w_{c}$ and $p_{h}$ have been analysed and will be discussed shortly.

## Results and discussion

The joint requirements and energy cost for these two characteristic motions were computed in MATLAB. The three parameters of interest, $l_{ow}$ , $l_{w}$ and $l_{2}:l_{3}$ , were cycled through all possible combinations within the ranges defined in Table 1. This resulted in a total of 132 unique link combinations.

**Figure 5.**
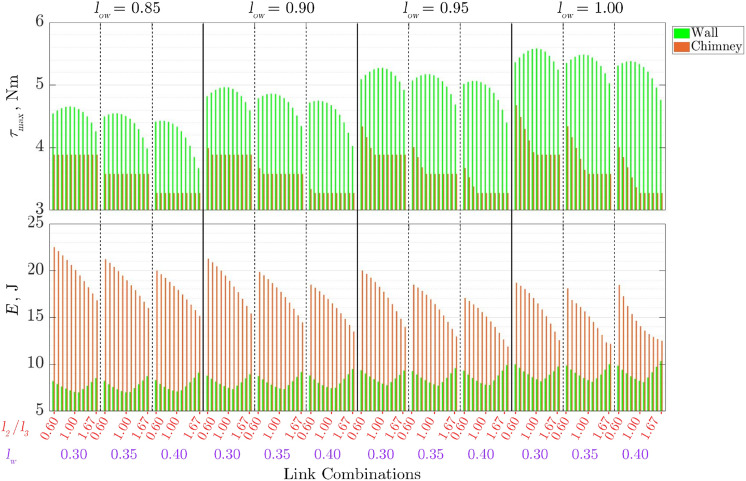
Energy and torque requirement for the wall transition and chimney walking.

The results for the peak torque requirement among any of the joints, $\tau_{\text{max}}$ , calculated from equation (2) and the total energy cost, *E*, calculated using equation (3) for both wall transition and chimney walking motions are shown in Figure 5. The labels on the top and bottom side of Figure 5 show the link value for $l_{ow}$ , $l_{w}$ and $l_{2}:l_{3}$ , respectively.

### Effect of parameters of interest

From Figure 5, increasing $l_{ow}$ results in a linear increase for both the energy and torque requirements for wall transition. For a fixed $l_{ow}$ , the maximum torque, $\tau_{\text{max}}$ , is when $l_{w}=0.3$ m and $l_{2}/l_{3}=0.9$ . On the other hand, the minimum torque, $\tau_{\text{min}}$ , is when $l_{w}=0.4$ m and the link ratio is at its largest, $l_{2}/l_{3}=1.67$ . The local minimum for *E* at any given $l_{w}$ is when $l_{2}/l_{3}=1.11$ , while the maximum is $l_{2}/l_{3}=1.67$ , coincident with the minimum $\tau_{\text{max}}$ . This situation arises as there is a high torque demand on each joint throughout the wall transition motion. It can be seen that *E* at $l_{2}/l_{3}=0.6$ is close to the maximum *E* as well. This is due to the larger joint velocities required when the link ratios are small.

For chimney walking, $\tau_{\text{max}}$ remains relative constant for any given $l_{w}$ . The required $\tau_{\text{max}}$ is observed to occur at the final pose and specifically on $\tau_{2}$ . The moment arm length between the femur joint, $q_{i,2}$ , and the foothold position is at its largest at the initial and final pose since the foothold lies in the middle of the motion path. While the foot force at both these pose is the same, the joint configurations are different. This difference yields a larger $\tau_{\text{max}}$ at the final pose compared to the initial pose. As $l_{ow}$ increases, having smaller link ratios would require larger $\tau_{\text{max}}$ . This is due to the maximum torque required by $\tau_{3}$ exceeding that of $\tau_{2}$ on the final pose.

### Effect of fixed parameters

**Figure 6.**
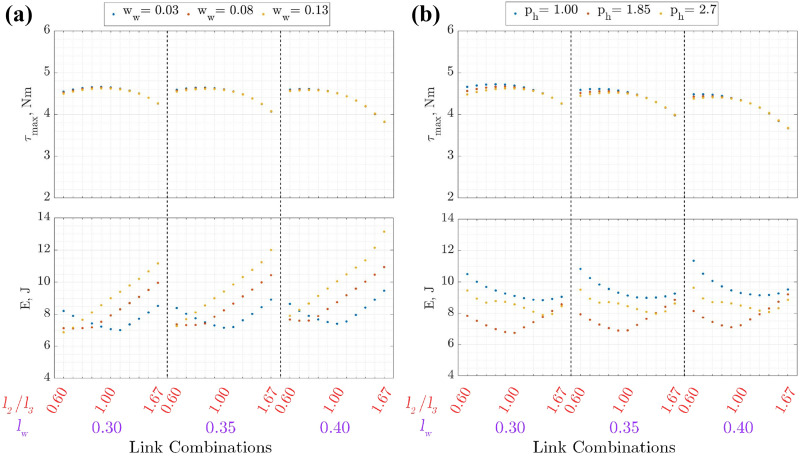
Torque and energy requirement at $l_{ow}=0.85$ m for wall transition with varying (a) $w_{w}$ and (b) $p_{h}$ .

**Figure 7.**
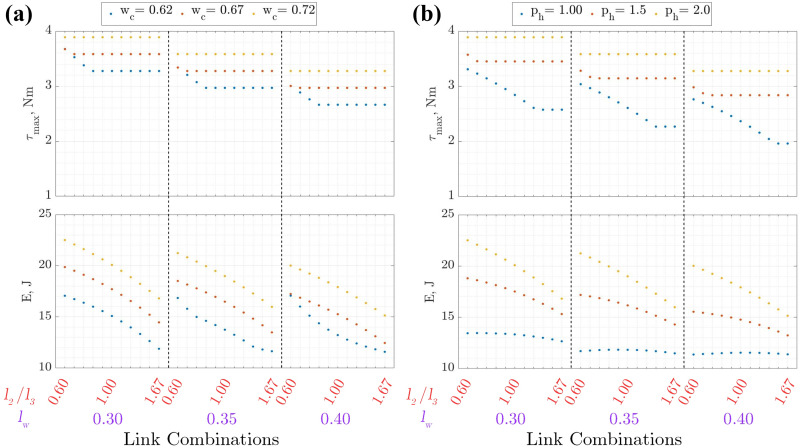
Torque and energy requirement at $l_{ow}=0.85$ m for chimney walking with varying (a) $w_{c}$ and (b) $p_{h}$ .

The effect of the environment parameters, namely $w_{w}$ , $w_{c}$ and $p_{h}$ , is shown in Figures 6 and 7. Only $l_{ow}=0.85$ m is shown here since the same trend is observed for different $l_{ow}$ .

For wall transition, the change in $w_{w}$ and $p_{h}$ has a small effect on $\tau_{\text{max}}$ . The instance at which $\tau_{\text{max}}=\tau_{3}$ occurs is in the middle of the transition ( $n_{d}=5$ ) on the right leg. Since the footholds and base position for the transition are interpolated linearly, the distance between the robot’s base and the right foothold becomes independent of $w_{w}$ . Hence, the joint angles for the right leg remain the same for different $w_{w}$ .

The result of the force distribution from equations (8) [Disp-formula disp-formula9-1729881419841537] to (10) changes according to the moment arm length to the left foothold, which is dependent on $w_{w}$ . However, only small changes in the forces are observed within the ranges evaluated for $w_{w}$ . Hence, $\tau_{\text{max}}$ remains approximately the same since both the joint angles and foot forces are largely similar. A similar trend on $\tau_{\text{max}}$ is observed for $p_{h}$ since the change in moment arm length is small which results in a deviation of only 0.25 Nm for the ranges evaluated.

The *E* generally increases with $w_{w}$ for larger link ratios. The increase in $w_{w}$ results in an overall increase in both the joint torque and velocity for the left legs, therefore increasing *E*. However, *E* for smaller link ratio is larger at small $w_{w}$ . This is because of the large change in $q_{2}$ at each $n_{d}$ , resulting in larger joint velocity. The effect of $p_{h}$ on *E* shows that the energy expenditure can be decreased by selecting $p_{h}=1.85$ . The minimum *E* also tends to be centred at $l_{2}:l_{3}=1.0$ .

For chimney walking, $\tau_{\text{max}}$ increases with $w_{c}$ within the ranges considered ( $0.62\text{m}\leq w_{c}\leq 0.72$ m). This is due to the increase in moment arm length between the joint and the foothold. The increase in $\tau_{\text{max}}$ leads to a similar response for *E*. A similar observation is seen, whereby $\tau_{\text{max}}$ increases with $p_{h}$ . $\tau_{\text{max}}$ for chimney walking occurs at the final pose since the moment arm length is the longest. At small $p_{h}$ , $\tau_{\text{max}}$ is on the tibia joint, while for large $p_{h}$ , it is on the coxa joint. This is due to the moment arm length between the contact point and the respective joints where the contact forces cause a larger moment around the tibia compared to the femur joint for smaller $p_{h}$ and vice versa.

The effect on the selection of $w_{w}$ and $p_{h}$ on $\tau_{\text{max}}$ for wall walking is negligible. Hence, the selection on joint requirements for wall walking can be based solely on $l_{ow}$ , $l_{w}$ and $l_{2}:l_{3}$ . On the other hand, the selection of joint requirements for chimney walking will heavily depend on $w_{c}$ , $p_{h}$ and $l_{2}:l_{3}$ as these parameters have a significant effect on $\tau_{\text{max}}$ . However, the effect of $l_{2}:l_{3}$ only needs to be considered for at larger $l_{ow}$ and small link ratios. This also means that joint requirements selected for chimney walking will be severely limited to the values used for $w_{c}$ and $p_{h}$ during the motion characterization.

From an optimization perspective on energy consumption, there is no global minimum for both wall transition and chimney walking. *E* for chimney walking is at a minimum when $l_{2}:l_{3}=1.67$ but this corresponds to a maximum for wall transition. Furthermore, *E* is highly dependent on the parameters $w_{w}$ , $w_{c}$ and $p_{h}$ . This means that optimizing the motion plan would yield better results as compared to optimizing the link parameters.

## Corin platform

**Figure 8.**
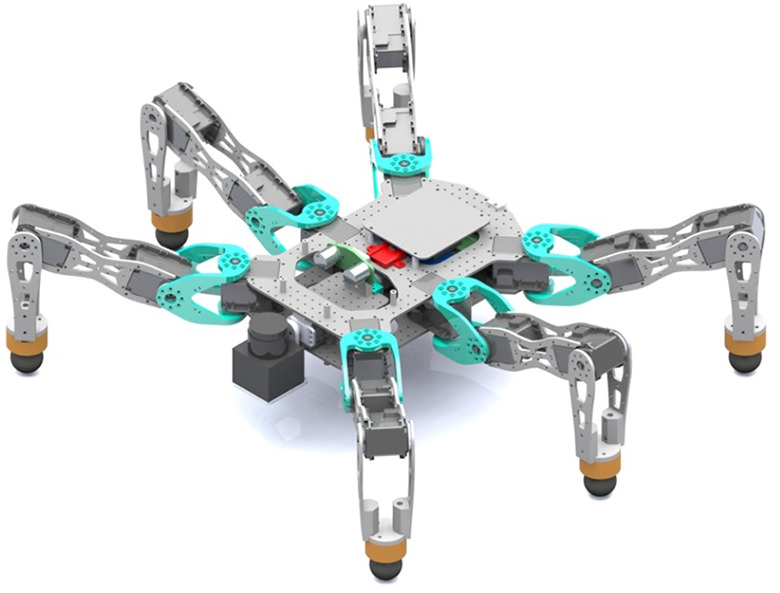
CAD image of Corin equipped with LiDAR and visual sensors.

This section briefly presents the mechanical design of the Corin hexapod, as shown in Figure 8, that is agile and versatile for multi-legged research in advanced motions.

### Hardware design

**Figure 9.**
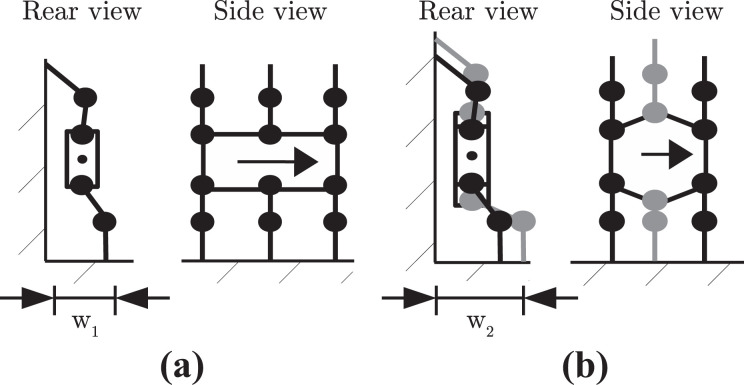
Comparison of rectangular with hybrid/hexagonal shape for sideways walking.

A rectangular body shape has been selected since this results in the smallest footprint width for wall and chimney walking compared to the hybrid or hexagonal shapes ( $w_{1}<w_{2}$ ). This is due to the middle pair of legs extending further out for the hybrid and hexagonal body shape, illustrated in Figure 9, for wall walking. Two additional actuators have been added on the body which enables sensors such as cameras or LiDAR to remain right-side up for upside-down operation. The legs were mounted to the body using the orientation similar to the DLR Crawler.^21^

The standard insect leg consisting of three DoFs has been employed for its larger workspace compared to parallel manipulators. The hip consists of two joints: the coxa, $q_{1}$ , and femur joint, $q_{2}$ . The axes of rotation for these two joints are perpendicular to each other. To obtain a symmetrical workspace about the sagittal plane, $q_{2}$ is mounted along the *x*-axis of $q_{1}$ . Each joint is actuated by a Robotics Dynamixel MX64 s for its high torque capability.

To enable force/position control, a three-axis capacitive force sensor has been attached to each foot enabling 3D contact force measurement. The rubber feet provide high friction and damping to reduce slipping and impact forces.

### Link parameter selection

Since the joint actuators were selected first, the link parameters were chosen to achieve objectives of ensuring that the joint torque requirement of the characteristic motions lies within the selected actuator constraint^16^ and maximizing the robot’s mobility.^18^

The use of *E* for selecting link length is not considered as there is no minimum that coincides for both wall transition and chimney walking. The joint torque constraint limits the parameter selection to $l_{ow}\leq 0.90$ m. To maximize mobility, the body footprint ( $l_{w}$ and $l_{bl}$ ) should be minimized,^21^ and the link length maximized.^22^ This corresponds to maximizing $l_{ow}$ while minimizing $l_{w}$ , which corresponds to 0.9 and 0.3 m, respectively. This results in a total leg length of 0.3 m ( $l_{2}+l_{3}$ ) for each leg.

Within the link ratio range for $l_{ow}=0.9$ m and $l_{w}=0.3$ m, any of the link ratios can be used as they meet the torque constraint. Hence, the decision was made to select $l_{2}:l_{3}=1:1$ since this link configuration has the maximum manipulability.^23^

**Figure 10.**
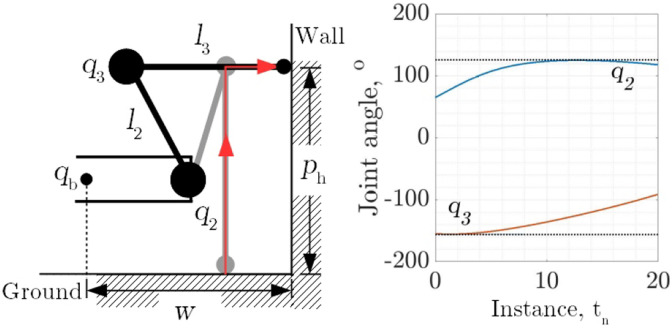
Joint angle requirements for moving the leg from ground to wall when the leg is tucked in. The red arrow line indicates the discretized instance, *n*, of the foot path.

Following on from the selection of the link ratio, the joint angles corresponding to the first wall contact foot position for wall transition have been identified to be $q_{2}=65{}^{\circ}$ and $q_{3}=-155{}^{\circ}$ . The translations and the resulting joint angles, both shown in Figure 10, set the required angles to be $q_{2}=126{}^{\circ}$ and $q_{3}=-156{}^{\circ}$ , respectively.

The length of $l_{1}$ has been selected to allow $q_{2}$ to meet its range of motion required. Next, $l_{bw}$ has been calculated from equation (1). Finally, $l_{bl}=0.23$ m has been selected for the leg’s work envelope to intersect each other for fault tolerant capabilities.^24^ Table 2 summarizes the technical specifications of Corin.

**Table 2.** System overview of the Corin hexapod.

Description	Value
Dimensions (nominal stance)	551 m × 0.622 m × 0.185 m (L × W × H)
Link lengths and weights	Coxa (*l*_1_): 0.060 m, 0.025 kg
	Femur ( $l_{2}$ ): 0.150 m, 0.280 kg
	Tibia ( $l_{3}$ ): 0.150 m, 0.09 kg
Weight	4.6 kg (off-board PSU)
Active DoF	20 (3 per leg, 2 for sensors)
Actuator type	Dynamixel MX-64AT (6.0 Nm)
Joint motion range	$q_{1}:\pm 48{}^{\circ}$ , $q_{2}:\pm 127{}^{\circ}$ , $q_{3}:\pm 160{}^{\circ}$
Actuator sensors	Position encoder (360° at 4096 cpr),
	Current sensor (4096 cpV),
	Voltage sensor (0–16 V)
Onboard sensors	Three-axis force sensor (80/100 N), IMU
Onboard computer	Odroid XU4 with Lubuntu 14
Controller rate	100 Hz

DoF: degree of freedom.

## Validation experiments

**Figure 11.**
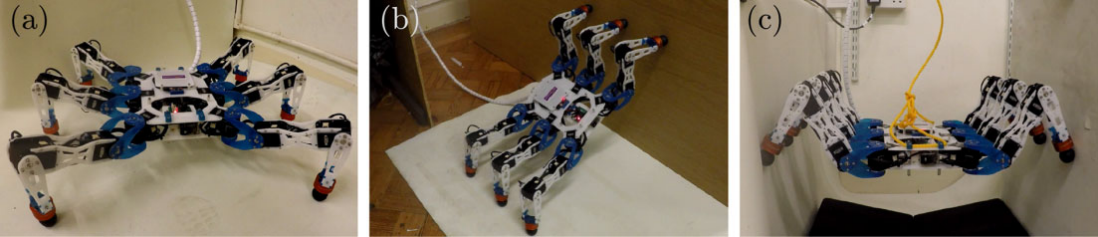
(a) Corin hexapod, evaluated for (b) wall transition and (c) chimney walking motions.

The Corin platform has been developed, both in terms of simulations and real implementation, as a vehicle to explore the ability of a hexapod of fairly conventional design to implement non-planar hexapod motions. In this initial investigation, the focus has been on the ability of the hexapod to adopt static poses and semi-static motions that are typically part of the transitions between floor and wall/chimney walking (see Figure 11). The limitations of this approach are understood; nevertheless, this quasi-static evaluation provides important basic information about the requirements on a hexapod that is intended to perform non-planar manoeuvres.

### Experimental configuration

A physical implementation of Corin has been developed and subjected to a program of testing in simple non-planar scenarios. The robot was powered through an external 12 V supply via an umbilical. This reduced the total weight of the vehicle, which was measured to be 4.6 kg. The environmental surfaces used in the experiments were either plain wood or rubber mats. The friction coefficients for these two surfaces combined with Corin’s rubber feet were estimated to be 0.83 and 0.87, respectively. This range is encompassed by the design analysis presented in the earlier section.

The robot was evaluated in terms of its capability to maintain static poses and execute statically stable motions for both wall and chimney walking. In the former case, the robot was positioned in selected transitional poses and the number of support feet was gradually reduced from six to three for a wall transition and from six to four for chimney walking. This shows that the robot is able to retain its static pose, in accordance with the design analysis presented earlier.

In each of the selected poses, the robot was required to translate 0.05 m along each of the three axes, with six legs in support. This ensures that non-planar movements can be achieved using discontinuous phase gaits. The motions were evaluated in open-loop mode, and so there was no active regulation of the body orientation. The joints were controlled using the actuator’s onboard position controller.

Each evaluation was repeated three times. Table 3 lists the parameters of the robot pose and leg positions, with respect to the leg frame. In the wall transition experiments, the robot’s pose was measured relative to the floor-wall boundary. For chimney walking, the robot was positioned in the middle of the chimney, and its static pose observed for a number of chimney widths, each differing from the previous width by 0.05 m.

**Table 3.** Parameters for wall transition and chimney walking motions.

No.	Wall transition		
	Robot’s pose [*y*, *z*, roll] (m, m, °)	Left leg stance [*x*, *y*, *z*] (m, m, m)	Right leg stance [*x*, *y*, *z*] (m, m, m)
1	[0.24,0.22,30]	[0.23,0,0.06]	[0.28,0,−0.04]
2	[0.15,0.34,60]	[0.27,0,0.04]	[0.29,0,−0.02]
3	[0.10,0.42,90]	[0.18,0,−0.06]	[0.33,0,0.10]
	Chimney walking		
No.	Chimney width *d* (m)	Leg stance [*x*, *y*, *z*] (m, m, m)	
1	0.62	[0.22,0,0]	
2	0.67	[0.24,0,0]	
3	0.72	[0.27,0,0]	

## Results

### Wall transition

**Figure 12.**
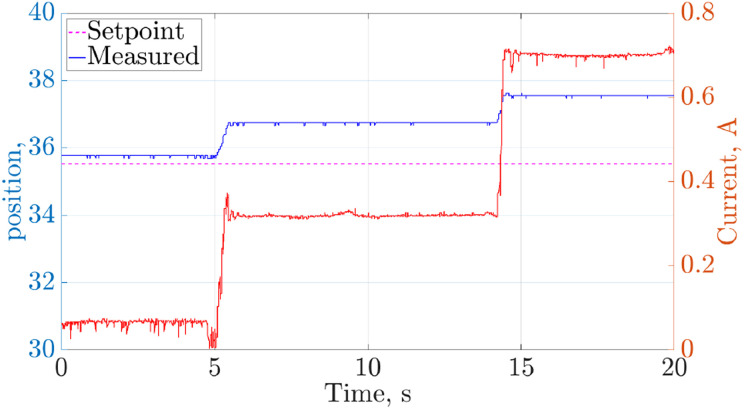
Position tracking and current of leg 4 femur joint when the number of support legs decreased during the transition pose.

Figure 12 shows the position tracking of leg 4; support legs were removed at times 5 s (leg 2), 10 s (leg 3) and 15 s (leg 6). The current drawn by leg 4’s femur (see Figure 2) servo is also shown in Figure 12.

It can be deduced that leg 3 provided limited support for the robot, as there was minimal change to leg 4’s joint position and current draw when it was lifted up at t=10 s. The change in current drawn by the femur and tibia servos was less than 0.06 A. On the other hand, there was a significant increase in the current draw when legs 2 and 6 were lifted off the ground at t=5 s and t=15 s, respectively. However, the increase was within the actuator’s limit of 4.1 A. The position tracking of the joint using the actuator’s encoder, which has a resolution of 0.088, degraded slightly by 2°, but this is considered unimportant.

**Figure 13.**
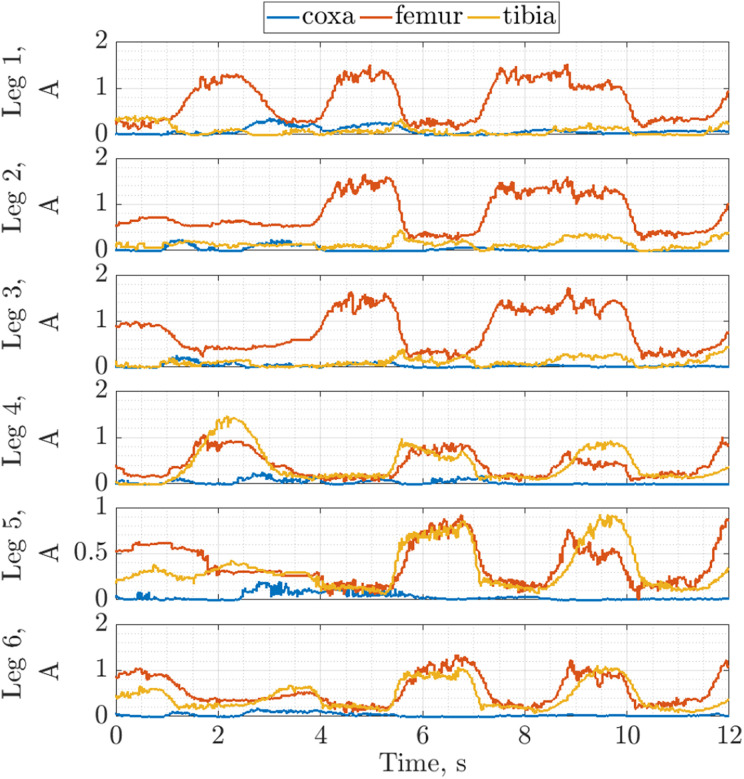
Joint current for all six legs executing motions representative of the wall transition motion.

For the evaluation of the motion, the robot tracked a continuous path with 3D translations (see supplementary video for static poses and semi-static motions). The currents drawn by all the robot joint actuators were well below the stall current limit (see Figure 13). Low PID gains were used with the joint controllers in the absence of higher level controllers to avoid large internal forces. This resulted in a slight degradation of position tracking with a maximum error of 4°, but this is considered to be well-within acceptable bounds. Hence, it can be concluded that the robot is able to execute the wall transition manoeuvre.

### Chimney walking

**Figure 14.**
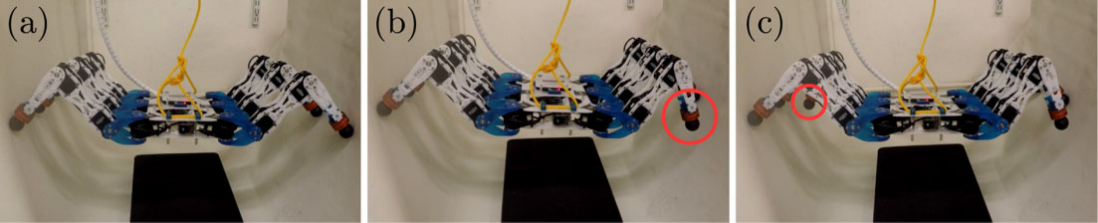
Corin maintaining static position by supporting itself between two walls with (a) six legs, (b) five legs and (c) four legs. The red circles indicate the leg removed from support.

In the chimney walking experiments, the Corin hexapod was able to maintain its position when legs 1 and 6 were lifted off the walls (Figure 14). It tilted forward slightly when leg 1 was lifted as the contact forces generated by the five remaining supporting legs caused a moment about the robot’s centre of the mass. Due to symmetry, the initial tilt was removed when leg 6 was lifted. The contact forces did not generate any noticeable moments since there was no observable change in the robot’s orientation.

**Figure 15.**
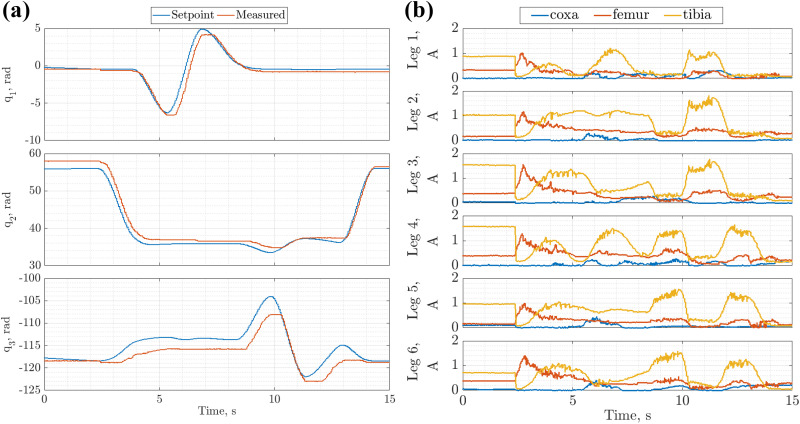
Joint tracking and current for motions representative of chimney walking motion. (a) Joint tracking for leg 4. (b) Joint current for all six legs.

Figure 15(a) and (b) shows the joint tracking and the current drawn by each of the servos controlling the robot’s leg 4 joints. Joints $q_{4,1}$ and $q_{4,2}$ tracked the set point closely, while $q_{4,3}$ had slightly poorer tracking performance with a maximum error of 4°. It can be seen that as the tracking error on $q_{4,3}$ increases as the current draw increases. As in the wall transition experiments where low PID gains were used, the current draw was within the actuator stall limit. Although there is a tracking error, the motion was observed to be relatively smooth, and the error can probably be compensated through the introduction of higher level controllers.

### Comparison with other approaches

Three approaches of characterizing joint torques that have been reported in the literature^8,13,25^ were applied to Corin. Table 4 shows the results obtain from these approaches. The result from the analysis presented earlier in this article is included for comparison.

**Table 4.** Comparison of joint torque using different characterization approaches.

	Approaches			
Peak torque (Nm)	1^25^	2^13^	3^8^	Proposed
$\tau_{2}$	4.91	3.79	3.10	3.89
$\tau_{3}$	2.46	1.90	1.01	4.97

The approaches from the literature are used to obtain torque-demand bounds for two-dimensional hexapod motion that is confined to a ground plane. The three different approaches all show that $\tau_{2}$ is smaller than $\tau_{3}$ . For two-dimensional movements, this is to be expected because $q_{2}$ is nearly always further away from the point of foot contact as compared to $q_{3}$ . This results in a longer moment arm and hence a larger $\tau_{2}$ requirement.^8^ However, the results of the analysis of this article show that $\tau_{2}$ is smaller than $\tau_{3}$ for both wall and chimney walking since in these situations, the foot contact forces do not act directly through $q_{3}$ . Unsurprisingly, this comparison shows that standard approaches to torque characterization are insufficient to establish torque demand when non-planar motions are executed and highlights the need for the more advanced analysis presented in this article.

A limitation of this work is that only static and semi-static manoeuvres are shown. However, these are precursor to the full manoeuvre which requires high-level controllers with force feedback and torque control.^20^ Since the robot here currently utilizes position controllers only, it is thus limited to only static and semi-static movements. Future work will address the development of the controllers necessary for achieving the full motion.

## Conclusion and future work

This article has presented a static 2D analysis of advanced hexapod manoeuvres, specifically wall and chimney walking. The analysis shows the relationship between link parameterization and joint torque demand. The joint range of motion has also been identified as part of this analysis. The analysis has been used to select the link parameters for the Corin hexapod, which generally assumes the design of well-established hexapods. The two forms of advanced motion have been validated experimentally with a real Corin hexapod. The work shows that hexapods of fairly standard design are capable of advanced manoeuvres, so long as suitable link length and joint actuators are properly selected. The ability of hexapods to execute such advanced manoeuvres enables them to explore areas previously deemed inaccessible.

It has been observed in the experiments that the use of position control leads to situations where the robot momentarily loses its balance. This can be addressed through the use of a high-level controller that distributes foot force equally (using torque and foot force feedback). Furthermore, the use of low-level controllers on each leg, such as impedance controller, allows robust operation on irregular surfaces, reducing the computation burden on the high-level controller.

## Appendix 1

### Final body pose for wall transition

The final body pose for wall transition is calculated knowing $q_{i\text{(init})}$ , $q_{i(\text{final})}$ and $w_{c}$ . Since leg 2 is in ground contact, the robot’s base height is expressed as

1A
$$q_{b(\text{final})}(z)=l_{w}/2+l_{2}\text{cos}(q_{2,2(\text{final})})+l_{3}$$

The distance between the robot’s base final position and the wall is expressed by

1B
$$y=l_{3}\text{sin}(q_{1,3(\text{final})})$$

The distance from the robot’s initial base to the wall is expressed by

1C
$$w=l_{w}/2+l_{2}\text{cos}(q_{1,2(\text{init})})+w_{c}$$

Finally, the robot’s base final position relative to the world frame is

1D
$$q_{b(\text{final})}(y)=w-y$$

### Final joint angles for chimney walking

The initial and final height of the robot’s base is expressed by

1E
$$q_{b(\text{init})}(z)=l_{3}-l_{2}\text{sin}(q_{1,2}(\text{init}))$$

1F
$$q_{b(\text{final})}(z)=q_{b(\text{init})}(z)+d_{z}$$

The vector from the femur joint to the foot at the robot’s final body pose is

1G
$$p_{\text{foot}}^{\text{coxa}}=\left[\begin{array}{c} w_{c}-l_{w}/2\\ d_{z}/2 \end{array}\right]$$

The final joint angles can then be calculated using inverse kinematics.
